# Interferon-Gamma Release Assay (Modified QuantiFERON) as a Potential Marker of Infection for *Leishmania donovani*, a Proof of Concept Study

**DOI:** 10.1371/journal.pntd.0001042

**Published:** 2011-04-19

**Authors:** Kamlesh Gidwani, Stephen Jones, Rajiv Kumar, Marleen Boelaert, Shyam Sundar

**Affiliations:** 1 Banaras Hindu University, Varanasi, India; 2 Cellestis limited, Chadstone, Victoria, Australia; 3 Institute of Tropical Medicine, Antwerp, Belgium; Lancaster University, United Kingdom

## Abstract

**Background:**

In areas endemic for visceral leishmaniasis (VL), a large number of infected individuals mount a protective cellular immune response and remain asymptomatic carriers. We propose an interferon-gamma release assay (IFN-γRA) as a novel marker for latent *L. donovani* infection.

**Methods and Findings:**

We modified a commercial kit (QuantiFERON) evaluating five different leishmania-specific antigens; H2B, H2B-PSA2, H2B-Lepp12, crude soluble antigen (CSA) and soluble leishmania antigen (SLA) from *L. donovani* with the aim to detect the cell-mediated immune response in VL. We evaluated the assay on venous blood samples of active VL patients (n = 13), cured VL patients (n = 15), non-endemic healthy controls (n = 11) and healthy endemic controls (n = 19). The assay based on SLA had a sensitivity of 80% (95% CI = 54.81–92.95) and specificity of 100% (95% CI = 74.12–100).

**Conclusion:**

Our findings suggest that a whole-blood SLA-based QuantiFERON assay can be used to measure the cell-mediated immune response in *L. donovani* infection. The positive IFN-γ response to stimulation with leishmania antigen in patients with active VL was contradictory to the conventional finding of a non-proliferative antigen-specific response of peripheral blood mononuclear cells and needs further research.

## Author Summary

Visceral leishmaniasis is caused by a parasite of the Leishmania species, but infection does not always lead to overt clinical disease. To detect infection, the Montenegro test or Leishmanin Skin Test (LST) is used along with serological markers. The LST is a test of the delayed-type hypersensitivity response read 48–72 hours after intradermal injection of leishmanin antigen. LST has many drawbacks, as complex administration and reading, boosting of anamnestic immune responses and difficult sourcing of GMP-compliant product and alternative tools for epidemiological research are badly needed. We evaluated whether a Interferon-γ Release Assay based on the QuantiFERON-TB test format, which was approved by the Food and Drug Administration (FDA) as a test for detecting latent *Mycobacterium tuberculosis* infection, could become an in vitro diagnostic aid for the measurement of cell-mediated immune reactivity against *L.donovani*. We obtained good results with one of five of the antigens we evaluated and confirm the potential of this assay.

## Introduction

Visceral leishmaniasis (VL) also known as kala-azar is the most severe form of leishmaniasis and is a chronic systemic disease that is fatal unless treated. The main clinical symptoms are an enlarged spleen and prolonged irregular fever [1]. Approximately 500,000 new cases of human VL occur annually according to WHO estimations. India, Nepal, Bangladesh, Sudan and Brazil harbour 90% of the worldwide reported VL cases. In 2005 the governments of India and Bangladesh embarked on a VL elimination program with the goal to reduce the incidence of disease below 1 per 10 000 per year (TDR 2005).

However, leishmanial infection does not always lead to overt clinical VL. Leishmanial DNA was detected by PCR in peripheral blood of subclinically infected patients [2] and promastigotes of *L.infantum* can be cultured from the buffy coat of asymptomatically infected blood donors [3]. The asymptomatic *L.donovani* carriers usually outnumber the number of clinical cases, with ratios varying between 4 to 1 in Bangladesh [4] and 10 to 1 in India and Nepal [5]. Asymptomatic infected persons were so far never targeted for treatment, as their role in disease transmission dynamics is poorly understood, and current drugs are too toxic to justify their use in otherwise healthy individuals. For the VL elimination initiative it will be critical though to document the trends in incident *L.donovani* infections in the next few years. Typically, serological methods as Direct Agglutination Test (DAT) and k39 ELISA or leishmanin skin testing (LST) have been used to document incident infection, but their respective value as markers of infection has not been firmly established. Serologic testing is generally assumed to detect more recent infection, but the length of time for which serology remains positive, and whether this differs between kala azar patients and asymptomatically infected individuals (as seems likely based on the magnitude of the titers), is not known with certainty.

LST is a marker of cell-mediated immunity and remains positive for many years after the initial infection. Moreover, in VL cases, LST is known to become negative during the anergic phase of acute VL, and to return to positive once the cell-mediated immune response is restored [6]. However, leishmanin antigen is not well standardized and there is clear variability in sensitivity and specificity from one antigen to another, and in one study, there was evidence of loss of antigen potency over time [7], [8]. A previous study found that the sensitivity of LST to detect cell-mediated immunity in cured VL patients was low in India [9]. Having to read the LST after 48 hrs doesn't make it a very practical test for field use. Nevertheless, markers of the cellular immune response hold promise; T cell proliferation is a possible outcome of T cell activation and the measurement of IFN-γ released by activated T cells refines assessment of cellular immune response. In vitro IFN-γ release assays (IFN-γRA) (e.g. QuantiFERON-TB Gold) have been developed to document latent *Mycobacterium tuberculosis* infection. This assay detects IFN-γ in whole blood stimulated with *Mycobacterium tuberculosis* specific peptide antigens with very good sensitivity and specificity [10], [11]. A similar assay based on leishmania antigen(s) might also be valuable for leishmaniasis. A recent study in Turkey explored the use of a modified QuantiFERON assay to detect the cell-mediated immune response against *L.infantum* [12]. We developed a novel IFN-γRA based on the QuantiFERON test system and evaluated its potential to detect *L.donovani* infection.

## Materials and Methods

### Study subjects

The study was conducted at Banaras Hindu University, Varanasi and the Kala-azar Medical Research Centre (KAMRC), Muzaffarpur, India. The study was approved by the Institutional Ethics Committee of Banaras Hindu University. Written informed consent forms were obtained from study subjects. Four different groups were consecutively recruited: 1) Active VL patients (n = 13); 2) Subjects cured from VL at least six months after the end of successful treatment (n = 15); 3) Healthy controls living in the endemic region with no prior history of kala azar (n = 19); and 4) Non-endemic Healthy Controls having no travel history to Bihar (n = 11). The inclusion criteria for active VL patients in this study were the presence of parasite in splenic aspirate confirmed by microscopy and having related clinical symptoms. The microscopic confirmation of parasite in splenic aspirate was performed by expert technicians. All cured VL subjects have negative microscopy at the end of successful treatment. No any clinical investigation was carried out as the part of this study. The information about patients's clinical diagnosis was available at KAMRC, Muzaffarpur. Subjects having fever history in past one month and age below 5 years were excluded from the study.

### Antigens

QuantiFERON ELISA kits, nil tubes (tubes with no added antigen) and precoated blood collection tubes with peptides covering **1) Leish A** [H2B peptides], **2) Leish B** [peptide pool of H2B and PSA-2] **3) Leish C [peptide pool of H2B and Lepp 12**] proteins, and Phytohaemagglutinin (PHA) mitogen tubes were kindly donated by Cellestis limited, Chadstone, Victoria, Australia. Nil tubes were used for addition of *L.donovani* lysates (Crude Soluble Antigen (CSA) and Soluble Leishmania Antigen (SLA), and Phosphate Buffered Saline (PBS) (no antigen).

H2B has a complete sequence of 111 amino acids. It has 100% homology with *L.infantum, L.major* and *L.donovani* and 80% homology with *L.braziliensis*. PSA-2 peptides were from *L. major* and *L. infantum*, and shared 74% homology with *L.donovani* and 68% with *L.tropica*. Lepp12 is 88 amino-acid nuclear phosphoprotein of *L. infantum* and has 100% homology with *L major* and 92% with *L.braziliensis*. The H2B and PSA2 peptide antigens were selected after the initial screening of a series of antigens that produced requisite sensitivity and specificity in pilot studies conducted in Turkey [12]. Lepp12 peptides were added to H2B peptides to investigate its potential in this assay as it has been described as a highly sensitive and specific antigen for VL [13]. The whole organism antigens were included as controls.

CSA from *L. donovani* strains was prepared in the Banaras Hindu University laboratory. Stationary-phase promastigotes (1×10^8^ cells/mL) were harvested and centrifuged at 4000 rpm for 20 minutes at 4°C. The pellet so obtained was washed four times with cold PBS. Pellet was resuspended in PBS containing protease inhibitors (Sigma) and repeatedly freeze (−80°C) thawed 4 times with each cycle lasting 1–2 hours. In the last cycle, the freezing was done overnight and then the lysate was thawed, centrifuged at 16000 rpm and supernatant was collected ,and stored in −80°C until use. Protein estimation was done using bicinonic acid (BCA) methods.

SLA was prepared as described elsewhere. [14]. Briefly 2×10^9^ stationary-phase promastigote were harvested and centrifuged at 4500 rpm for 20 minutes to obtain the pellet. Pellet so obtained was washed thrice with cold PBS and then resuspended to 10 mM TRIS-HCl, 1 mM EDTA (pH8.0) 1.6 mM PMSF and 50 µg/mL leupeptin to obtain a concentration of 2×10^9^ parasites/mL. Now it was sonicated 4–5 times for 15 seconds at 10 Hz and centrifuged at 27000× g for 30 minutes at 4°C. After removing the lipid layer from the surface of supernatant, remaining solution were ultracentrifuged at 100000× g for 4 hrs at 4°C. The supernatant so obtained was stored at −80°C until use. Protein was estimated using BCA method. PHA and PBS were used as positive and negative controls, respectively.

### Collection of blood and Interferon-gamma Release Assay (modified QuantiFERON)

QuantiFERON tubes contain heparin and vacuum to draw 1 ml of venous blood. Blood was collected for each antigen tested and for positive (PHA) and negative (PBS) controls. Two additional nil tubes were drawn for addition of SLA and CSA at a concentration of 4.2 µg/mL and 2.5 µg/mL respectively. After adding antigens and thoroughly mixing, tubes were incubated upright in 37°C incubator for 20 hrs without humidity and CO_2_. After incubation and centrifugation at 2000 × *g* for 10 min, supernatant plasmas were collected and stored at −20°C for IFN-γ analysis.

### Measurement of IFN-γ

IFN-γ level in proliferated plasma was measured using the QuantiFERON-ELISA kit using manufacturer's instructions as described elsewhere [15]. A 4 point standard curve of known amount of IFN-γ in International Unit per ml (IU/ml) was used to determine the level of IFN produced in response to different leishmania antigens. Antigen-specific IFN-γ levels (expressed in IU/mL) produced in response to leishmania antigen stimulation were determined by subtracting background levels measured in the non-stimulated (PBS) samples. The calculation and interpretation of result was done using software provided by the manufacturer (Analysis Software v1.51 Cellestis Limited.) as follows: positive if the IFN-γ concentration in either of the antigen wells was >0.2 IU/mL; this cutoff was determined by ROC analysis of previous data and selecting the cut-off with highest sensitivity (83%) and specificity (100%) [12].

### Statistical analysis

The Mann-Whitney U-test was used for statistical analysis between two groups in the IFN-γRA. These statistical analyses were performed using SPSS software (version 18). Statistical difference was considered significance if p value was <0.05. The sensitivity of the IFN-γRA to detect the cell-mediated immune response was taken as the percentage positive in the post-treatment series (cured VL); while specificity was given by 1 minus positivity in non-endemic healthy control (Table 1).

**Table 1:** Number (%) of subjects with positive IFN-γ responses in IFN-γ RA using different leishmania antigens.

Study Group	Leish A (H2B)		Leish B (H2B+ PSA2)		Leish C (H2B+ Lepp12)		CSA		SLA		Mitogen	
	N	%	N	%	N	%	N	%	N	%	N	%
NEHC (n = 11)	0	0	1	9	2	18	7	63.6	0	0	11	100
*Specificity* *		100		91		82		36.4		100		-
EHC (n = 19)	2	10.5	4	21	2	10.5	13	68.4	5	26.3	19	100
Active VL(n = 13)	7	53.8	7	53.8	8	61.5	10	77	11	84.6	13	100
Cured VL (n = 15)	7	46.6	6	40	8	53.3	13	86.6	12	80.0	15	100
*Sensitivity*		46.6		40		53.3		86.6		80.0		

NEHC = Non endemic healthy control, VL = Visceral leishmaniasis, EHC = Endemic healthy control.

*Specificity expressed as 1 minus (number positive/n) (%).

## Results

The number and percentage of subjects yielding positive IFN-γ responses in IFN-γRA assay using 5 different leishmania antigens for the four study groups are shown in Table 1. All subjects in all study groups gave positive IFN-γ responses after stimulation with PHA, a non-specific activator of T cells, indicating all subjects were immunocompetent. The assay was positive after stimulation with H2B, H2B-PSA2, H2B-Lepp12 peptides, CSA and SLA in 0%, 9%, 18%, 63.6%, and 0% of the non-endemic healthy controls (n = 11), 10.5%, 21%, 10.5%, 68.4% and 26.3% of endemic healthy controls (n = 19), 46.6%, 40%, 53.3%, 86.6% and 80% of the cured subjects (n = 15), and 53.8%, 53.8%, 61.5%, 77.0%, 84.6% of active VL patients (n = 13), respectively. Highest sensitivity (80% [95% CI = 54.81–92.95]) and specificity (100% [95% CI = 74.12–100]) for a single antigen preparation was found using SLA.

**Figure 1:**
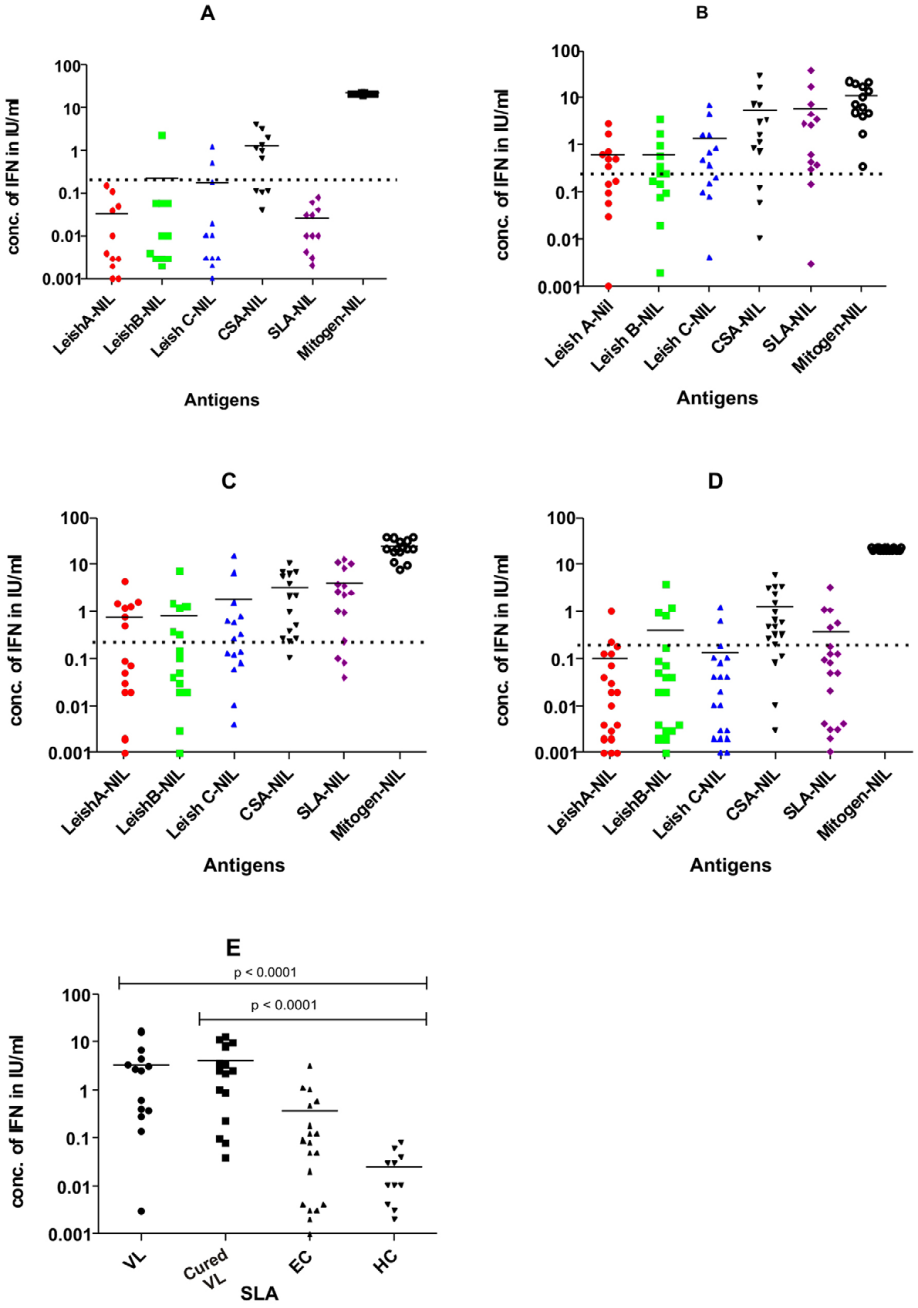
IFN-γ level in Proliferated Blood in different subject groups. IFN-γ (IU/mL) production by peripheral blood stimulated with Leish A (H2B), Leish B (H2B-PSA2), Leish C (H2B-Lepp12) peptides, crude soluble antigen (CSA) and soluble leishmania antigen (SLA) after subtracting the value of the No Antigen control (PBS) in (A) Non Endemic Healthy Controls (n = 11); (B) Active Visceral leishmaniasis (n = 13); (C) Cured VL , 6 month after successful treatment (n = 15); (D) Healthy endemic control subjects (n = 19) using whole blood IFN-γRA. Dotted lines represent the assay cutoff (0.2 IU/mL); (E) Comparison of IFN-γ levels (nil value subtracted) for different subject groups using SLA.

The mean IFN-γ level against different antigens in the proliferated blood of four different study groups has been shown in Figure 1. The mean concentration of IFN-γ against SLA treatment in active VL, cured VL, HEC and NEHC was 5.92, 4.0, 0.35 and 0.02 IU/ml respectively (Figure 1). Twelve of the 15 cured subjects with a history of kala azar showed SLA-specific IFN-γ production. However the SLA specific IFN-γ production in 11 of the 13 individuals with active VL disease was a surprising finding. In order to show IFN-γ release was occurring in vitro in response to SLA, we replaced the plasma of 5 VL subjects with that of NEHC individuals before treatment with SLA, and observed again production of IFN-γ above basal level (data not shown).

## Discussion

This is the first report on a whole blood Interferon-γ release assay (modified QuantiFERON) as a potential marker of *L.donovani* infection. We hope it may contribute to the development of better markers for infection and to understanding T-cell immune responses in the high endemic foci of VL in India (Bihar). We set out to identify an antigen that was highly specific (i.e. not stimulating any IFN-γ release in NEHC individuals who were never exposed to *L.donovani*) and maximally stimulated IFN-γ release in whole blood of cured VL patients sampled 6 months after cure. Among all the antigens tested in the different subject groups, SLA showed a specificity of 100% as none of the NEHC subjects were IFN-γ positive (Table 1). Leish A and Leish B also showed good specificity (100% and 91%), however their sensitivity was low compared to the SLA. CSA showed very low specificity (36.4%) compared to other antigens. CSA contains the membranous part of the leishmania promastigote which may act as mitogen leading to positive IFN-γ responses in NEHC group. Lepp12, which is nuclear phosphoprotein of *L. infantum*, has previously been described as good marker for diagnosis and prognosis of VL [13]. In our study Lepp12 showed low sensitivity and specificity (53% and 82% respectively) and also 10.5% of EC was IFN-γ positive with Lepp12. Its addition to H2B peptides did not yield major improvement in sensitivity and specificity. A recent report by Turgay et al [12] found a good sensitivity of a similar assay with the H2B antigen. However, in Turkey VL is caused by *L.infantum*, not *L.donovani*. The H2B sequence in *L.donovani* is homologous with that for *L.infantum* and hence similar sensitivity to that obtained in Turkey study was expected in our study. The apparent lower sensitivity of H2B found in this study may be due the different length of time between cure and drawing of samples for IFN-γRA. The cured VL patients in the Turkish study were sampled 3–17 years after treatment whereas in our study a sample was taken at 6 months.

This study was designed as a pilot evaluation of a prototype diagnostic test and used a limited number of purposefully selected individuals [16], hence further validation is required on a larger number of subjects. Moreover, an assay detecting 80% of cured VL patients will not necessarily detect asymptomatic cases with a distinct cell-mediated immune response with a similar frequency.

A surprising finding in our study was the production of IFN-γ in active VL upon stimulation with leishmanial antigen. Active VL subjects are immune-suppressed and their Peripheral blood mononuclear cells (PBMC) don't proliferate nor give IFN-γ response after leishmania antigen challenge in other studies [17], [18], [19]. In our study, all antigens tested showed positive IFN-γ responses in active VL with highest value observed with the SLA (84.6%). Even there was IFN- γ production after replacement of plasma (to exclude effect of anti- leishmanial antibodies and immune-complex mediated production of IFN- γ) suggesting that there was antigen driven IFN- γ production in the IFN-γRA. Since this IFN-γRA approximates the *in vivo* condition and obviates the need to purify the mononuclear cells, a possible explanation is that preparation of PBMC may remove some factor which has a positive effect on IFN-γ production in active VL.

A valid marker of *L.donovani* infection is crucial for intervention studies on vaccine or vector control and for monitoring of ongoing transmission in endemic areas, and such a tool should moreover be acceptable, robust and user-friendly. The whole process of test execution was very simple in the sense that i) venous blood has been directly collected in the special QuantiFERON collection tubes, ii) antigens were added in the laboratory and tubes were incubated at 37°C for 20 hrs, iii) supernatant was collected to analyse the IFN-γ by a one-step commercially available ELISA kit. So this IFN-γRA assay is as acceptable to the study population as any other test requiring venous blood draw and is quite simple to conduct in a regular research laboratory. It has therefore potential as a marker in future epidemiological research if its validity is confirmed.

In conclusion, an IFN-γRA based on SLA showed a potential as marker of *L.donovani* infection and should be validated further on a larger series of subjects. Furthermore, the cellular source of IFN-γ released in acute stage VL patients needs to be identified in future studies.

## Supporting Information

Figure S1 STARD checklist.
(0.05 MB DOC)
